# Defense Mechanisms of Two Pioneer Submerged Plants during Their Optimal Performance Period in the Bioaccumulation of Lead: A Comparative Study

**DOI:** 10.3390/ijerph15122844

**Published:** 2018-12-13

**Authors:** Dian Li, Linglei Zhang, Min Chen, Xiaojia He, Jia Li, Ruidong An

**Affiliations:** Institute of Ecology and Environment, State Key Laboratory of Hydraulics and Mountain River Engineering, College of Water Resource & Hydropower, Sichuan University, Chengdu 610065, China; lidian_1994@163.com (D.L.); chenm1004@gmail.com (M.C.); hexiaojia@scu.edu.cn (X.H.); lijia@scu.edu.cn (J.L.); anruidong@scu.edu.cn (R.A.)

**Keywords:** *Ceratophyllum demersum* L., *Hydrilla verticillate* (L.f.) Royle, bioaccumulation, defense mechanism

## Abstract

*Ceratophyllum demersum* L. and *Hydrilla verticillata* (L.f.) Royle, two pioneer, submerged plants, effectively remove heavy metals from contaminated water. The present work evaluates the bioaccumulation and defense mechanisms of these plants in the accumulation of lead from contaminated water during their optimal performance period. *C. demersum* and *H. verticillata* were investigated after 14 days of exposure to various lead concentrations (5–80 μM). The lead accumulation in both *C. demersum* and *H. verticillata* increased with an increasing lead concentration, reaching maximum values of 2462.7 and 1792 mg kg^−1^ dw, respectively, at 80 μM. The biomass and protein content decreased significantly in *C. demersum* when exposed to lead. The biomass of *H. verticillata* exposed to lead had no significant difference from that of the controls, and the protein content increased for the 5–10 μM exposure groups. The malondialdehyde (MDA) content and superoxide dismutase (SOD), peroxidase (POD), and polyphenol oxidase (PPO) activities were much higher in *C. demersum*, suggesting considerable damage from lipid peroxidation and sensitivity to lead stress. Enzyme inhibition and inactivation were also observed in *C. demersum* at high lead concentrations (40–80 μM). The excellent growth status, low damage from lipid peroxidation, and high activity of catalase (CAT) and phenylalanine ammonia-lyase (PAL) observed in *H. verticillata* illustrate its better tolerance under the same lead stress.

## 1. Introduction

As one of the major heavy metals, lead (Pb) has been an increasing concern for researchers because of its high toxicity, prevalent existence, persistence, and ubiquitous distribution [1]. Pb can have a negative impact on the morphology and growth status of plants [2], damage human health, and it does not have a beneficial effect on organisms [3]. Phytoremediation is an efficient, cost-effective, environmentally friendly method to address heavy-metal pollution with aesthetic value [4,5,6]. More than 500 species of plants have been documented to have an excellent ability to absorb extremely high levels of heavy metals [7,8]. Aquatic macrophytes act as important primary producers in aquatic ecosystems and possess considerable accumulation ability. Submerged plants possess a higher ability for the removal of heavy metals from wastewater than floating-leaved plants and emerged plants because they have a greater surface area and more biomass for the heavy metal accumulation [6,9]. The efficiency of phytoremediation greatly depends on the selection of an appropriate plant species for a particular heavy metal [10]. Thus, choosing a suitable submerged plant with high heavy metal accumulation ability is important for effective phytoremediation. According to Tangahu et al. [11], hyperaccumulators should survive despite the contaminant amounts they absorb. Two factors should be taken into consideration when selecting a plant for phytoremediation, i.e., its metal accumulation ability and metal tolerance characteristics.

Two submerged plant species were chosen for the experiments: *Ceratophyllum demersum* L. (Ceratophyllaceae family) and *Hydrilla verticillata* (L.f.) Royle (Hydrocharitaceae family). We selected these species for the following reasons: (1) They are cosmopolitan species distributed in temperate regions around the world, can adapt to many environmental conditions, and can be quickly cultivated in warmer regions [12,13,14], which means they can be used in phytoremediation more widely and economically. (2) Both species are among the most studied aquatic plants applied for phytoremediation and have been proven to possess excellent potential to accumulate various heavy metals from wastewater [15,16,17,18,19]. However, thus far, no experiments have been conducted to determine which of these two high-potential accumulator plant species possesses higher heavy metal accumulation ability under the same circumstances, and no comparison has been made to explore the similarities and differences in their defense mechanisms, especially during their optimal performance period.

Plants applied for phytoremediation should exhibit high and efficient absorption during their optimal performance period. According to previous studies, a period of 12–15 days has been proven to be an optimal performance period for plants treated with heavy metals [18,20,21,22,23,24]. Aquatic plants absorb considerable amounts of heavy metals but are not heavily poisoned after approximately 14 days of heavy metal exposure. For longer treatment times, the heavy metal accumulation in plants and the enzymes related to defense mechanisms may decrease, which have been proven in *C. demersum* and *H. verticillata* [25,26]. Therefore, 14 days is an optimal performance period for both species exposed to heavy metals and is also a representative period to reflect the bioaccumulation and tolerance characteristics of the plants.

As both species are pioneer plants with good accumulation ability, the defense mechanisms of these plants should be taken into consideration in the screening of plants for phytoremediation. This study explores the bioaccumulation and tolerance characteristics of *C. demersum* and *H. verticillata* during their optimal performance period and aims to determine which aquatic plant is a more efficient phytoremediator of Pb under various lead concentrations and how their defense mechanisms react to lead stress. In the present work, the comparisons of the defense mechanisms of these plants include the growth status, protein content, lipid peroxidation and antioxidant systems, etc. The performance of heavy metal accumulation ability and the defense mechanisms of plants during their optimal period could provide a criterion for the screening of hyperaccumulators.

## 2. Materials and Methods

### 2.1. Plant Materials and Treatment Conditions

*C. demersum* and *H. verticillata* without roots were collected from a plant market in Chengdu, China. After successively cleaning the plants with tap water and de-ionized water, the two plant species were placed in a 10% Hoagland nutrient solution [27] for a week to acclimatize them to the lab environment (115 μmol m^−2^s^−1^ light with a 12-h photoperiod at 25 ± 2 °C). *C. demersum* and *H. verticillata* (8 g per tank) were treated in 5-L tanks filled with 3 L of different concentrations of Pb (5, 10, 20, 40 and 80 μM) in 10% Hoagland nutrient solution for 14 days. The initial concentrations of lead were determined on the basis of the characteristics of lead-contaminated water determined in previous studies. Plants treated without Pb served as the control groups.

### 2.2. Quantification of Lead Bioaccumulation

To comprehensively compare the accumulation abilities of the two plant species, the whole plants were compared instead of differentiating various tissues. *C. demersum* is difficult to separate into leaves and stems. The plant samples were thoroughly washed and dried at 75 °C and then digested in HNO_3_:HCl (4:1, *v*/*v*). The lead content in the digested plants was determined using an atomic absorption spectrophotometer (Purkinje General Instrument Co., Ltd., Beijing, China) at 283.3 nm and is expressed as mg kg^−1^ dw.

### 2.3. Analysis of plant growth

The plant biomass was determined by the fresh weight of the harvested plants. The protein content was estimated according to the method of Sedmak and Grossberg [28] and expressed as mg g^−1^fw.

### 2.4. Lipid Peroxidation and Enzyme Activity Assays

All of the biochemical assays were conducted at 4 °C. Approximately 500 mg of fresh plants was gathered, ground as a chilled tissue homogenate at 60 Hz for 75 s and then extracted with 5 mL of 20 mM sodium phosphate buffer (pH 7.0) containing 0.5 mM EDTA and 0.15 M NaCl. The homogenate was centrifuged at 15,000× *g* for 5 min at 4 °C, and the supernatant was used for the antioxidant enzyme activity analyses.

Lipid peroxidation was estimated from the malondialdehyde (MDA) content following the slightly adjusted method of Heath and Packer (1968). The superoxide dismutase (SOD) activity was measured via the inhibition of nitroblue tetrazolium (NBT) following the method of Beauchamp and Fridovich [29]. The catalase (CAT) activity was assessed by measuring the decomposition of hydrogen peroxide according to the method of Aebi [30]. The peroxidase (POD) activity was measured following the method of Zhang et al. [31]. The phenylalanine ammonia-lyase (PAL) and polyphenol oxidase (PPO) activities were measured based on the methods described by Hahlbrock and Ragg [32] and Gao et al. [33], respectively. All enzyme activity is expressed as units g^−1^ fw.

### 2.5. Statistical Analysis

SPSS 17.0 (IBM Inc., Chicago, IL, USA) was used to perform the statistical analyses, and all experiments were repeated three times. The homogeneity assumptions for variance and normal distribution were tested with Levene’s test and Kolmogorov-Smirnov test, respectively. After the confirmation of these assumptions, one-way ANOVA was applied. For the F values, the degrees of freedom between and within groups were 1 and 4, respectively, and the total degrees of freedom was 5. Duncan’s multiple range test (DMRT) for post hoc multiple comparisons (*p* < 0.05) was also applied to assess the differences between treatments for each kind of plant and between different species of plants for each lead concentration.

## 3. Results and Discussion

### 3.1. Lead Accumulation

As shown in Table 1, the amount of lead accumulated by *C. demersum* and *H. verticillata* significantly depended on the lead concentration present in the nutrient solution (F = 359.723, *p* < 0.05, η^2^ = 0.993; F = 869.689, *p* < 0.05, η^2^ = 0.997). The level of lead accumulation in both plant species continued to increase with the increasing lead concentration, and the maximum lead accumulation in *C. demersum* and *H. verticillata* was 2482.7 mg kg^−1^ dw and 1792.0 mg kg^−1^ dw, respectively, for the 80 μM treatment. For each lead concentration, the lead accumulation amounts were different for the two plant species (*p* < 0.05). *C. demersum* had a better lead adsorption capacity.

Despite the controversial definition of ‘hyperaccumulator’ [34,35], both *C. demersum* and *H. verticillata* exhibited great potential to accumulate significant amounts of Pb, which further verifies the findings of previous studies [15,16,17,18,19]. Obviously, *C. demersum* possesses a stronger ability to absorb lead. The difference in the lead accumulation ability of these two species may be related to the fractal dimensions (FDs), which indicate that the spatial occupation and utilization of the leaves of *C. demersum* are greater than those of *H. verticillata* [36]. The results are consistent with the findings of Li et al. [9] that a higher surface area enables higher uptake of heavy metals. The lead accumulation in both plant species exhibited an increasing tendency with increasing lead concentration. In the 10 μM and 80 μM exposure groups, the lead accumulation in *C. demersum* was 4.9 times and 1.4 times, respectively, that in *H. verticillata*. The lead accumulation in *C. demersum* exhibited a logarithmic increase with the increasing lead concentrations, with R^2^ = 0.973. The increasing lead accumulation in *H. verticillata* was well fitted by the quadratic polynomial model with R^2^ = 0.984. This phenomenon suggests that as lead levels increase, the difference in the lead accumulation ability of these two species may further narrow. Speculations can be made that under higher concentrations of lead, the difference in FD may no longer play as important of a role in lead accumulation as that under lower concentrations, but the different defense mechanisms of plants instead become the governing factors. Thus, the defense mechanisms of plants should be further analyzed.

### 3.2. Growth Status

Biomass and protein content are good indicators of the growth performance of plants during lead exposure. The lead concentration had a significant influence on both the biomass and protein content of *C. demersum* (F = 50.564, *p* < 0.05, η^2^ = 0.955; F = 83.507, *p* < 0.05, η^2^ = 0.972, respectively) and *H. verticillata* (F = 3.365, *p* < 0.05, η^2^ = 0.584; F = 110.028, *p* < 0.05, η^2^ = 0.979, respectively).

**Figure 1:**
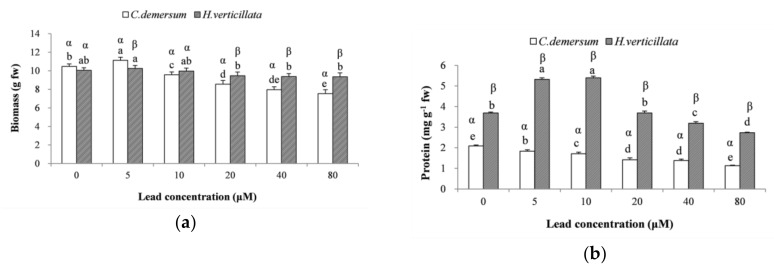
Effects of lead toxicity on (**a**) biomass and (**b**) protein content in *Ceratophyllum demersum* and *Hydrilla verticillata.* All values are the mean of triplicate measurements ± SD (*n* = 3). ANOVA was considered significant at *p* < 0.05. Different Roman letters (a–f) and different Greek letters (α, β) indicate significant differences between concentrations for a plant species and significant differences between plant species for a concentration, respectively (Duncan’s multiple range test (DMRT), *p* < 0.05).

Figure 1 shows that the biomass of both species showed slight increases at 5 μM and then continually decreased with the increasing lead concentration. Because the plants were not greatly affected by lead toxicity after 14 days of exposure, the minimum biomass was observed with 80 μM Pb and was 72.0% and 93.1% of the levels in the controls for *C. demersum* and *H. verticillata*, respectively. The reduction in the biomass in plants treated with high lead concentrations verified the toxicity of Pb. These macroscopic effects are mainly a result of toxic effects on cell constituents, water status, mineral nutrition, photosynthesis, and respiration [37]. The slight increase in biomass with low levels of Pb suggest that low doses of Pb may encourage plant growth, which was also observed in another study [38]. The biomass of *H. verticillata* treated with varying lead concentrations exhibited no significant differences compared to that of the controls (*p* > 0.05). The biomass in the 80 μM exposure group was not significantly different from that of the controls even though the lead content in *H. verticillata* was 1792.0 mg kg^−1^ dw; the biomass was also higher than that of *C. demersum* exposed to 20 μM Pb, with a lead accumulation of 1653.8 mg kg^−1^ dw. This phenomenon indicates the high growth status of *H. verticillata* despite the high lead content in the plants.

Compared with the biomass response, the protein content response was more sensitive to Pb. A significant decrease in the protein content was observed in *C. demersum*, and the minimum value at 80 μM Pb was 54% of the value in the controls. It has already been noted that protein synthesis can be disturbed as a result of the harmful effects of heavy metals [39], and high concentrations of Pb may cause a reduction in the protein pool [37,40]. For *H. verticillata*, the protein content increased at low doses (5 and 10 μM) and then sharply decreased at high doses (20~80 μM). The highest and lowest protein contents occurred at 10 and 80 μM Pb and were 146% and 74% of the levels in the controls, respectively. The results indicate that protein synthesis in *H. verticillata* was promoted in the presence of low levels of Pb and inhibited at high levels. This increase in the protein content may result from increased synthesis of defense proteins to resist lead toxicity, especially proteins that play a role in maintaining the redox status [37,41]. However, Mishra et al. [42] observed an induction in the protein content in *C. demersum* exposed to 1–50 μM lead for 2 days. The specific reason for the increase in the protein content of *C. demersum* was not observed in the present study, possibly because longer durations resulted in an increase in proteolytic activity induced by metal toxicity [43]. Therefore, the synthesis of defense proteins in *C. demersum* can be activated under a shorter exposure time (i.e., 2 days) and might be disturbed after a longer lead exposure time, such as 14 days. In contrast, the increased protein content observed in *H. verticillata* under the low lead concentrations at the 14th day seems to indicate its higher lead tolerance potential.

### 3.3. Lipid Peroxidation Products and Antioxidant Enzymes

**Figure 2:**
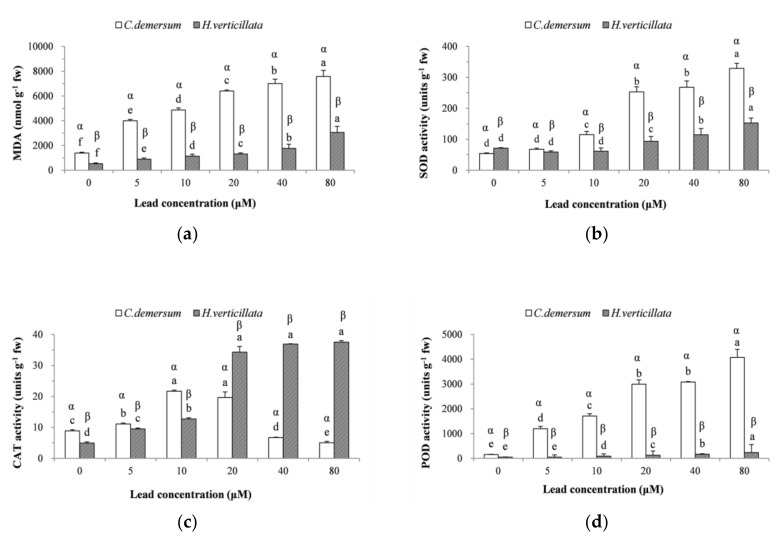
Effects of lead toxicity on (**a**) malondialdehyde (MDA), (**b**) superoxide dismutase (SOD), (**c**) catalase (CAT), and (**d**) peroxidase (POD) in *C. demersum* and *H. verticillata.* All values are the mean of triplicate measurements ± SD (*n* = 3). ANOVA was considered significant at *p* < 0.05. Different Roman letters (a–f) and different Greek letters (α, β) indicate significant differences between concentrations for a plant species and significant differences between plant species for a concentration, respectively (DMRT, *p* < 0.05).

Previous studies have reported that Pb induces oxidative stress by increasing the production of ROS (Reactive Oxygen Species) in plants [44], and then antioxidant systems are naturally activated to remove the toxicity of ROS [45]. MDA, which is a thiobarbituric acid reactive substance, is produced by lipid peroxidation and is widely used to indicate the extent of oxidative stress [24]. As MDA is not advantageous for plant growth, a high MDA content indicates that the stress tolerance of a plant is weak. The MDA content in both *C. demersum* and *H. verticillata* (Figure 2a) was significantly affected by the lead concentration (*C. demersum*, F = 230.744, *p* < 0.05, and η^2^ = 0.990; *H. verticillata*, F = 291.855, *p* < 0.05, and η^2^ = 0.992) and exhibited the same increasing trends corresponding to the increase in the lead concentration. Similar results were also reported by Dogan et al. [17] for *C. demersum* exposed to Cd and Pb and Song et al. [18] for *H. verticillata* exposed to Ni. The MDA content in *C. demersum* and *H. verticillata* reached a maximum at 80 μM and was 543% and 583%, respectively, of the levels in the controls. Furthermore, the MDA content exhibited a strong positive correlation with lead accumulation in both *C. demersum* (R^2^ = 0.945) and *H. verticillata* (R^2^ = 0.979), which further indicates that lipid peroxidation in plants is directly associated with lead stress. For each lead concentration, the MDA content in *H. verticillata* was much lower than in *C. demersum*—approximately 20–40% of the levels in *C. demersum*—indicating that *H. verticillata* suffers lower damage from lipid peroxidation under the same lead concentration.

SOD is the first line of defense in the antioxidant system and can alleviate the toxic effect of O^2−^ in plants [46]. O^2−^ is one of the major ROS produced in cells in the physiological state [10]. The significant changes in SOD activity (*C. demersum*: F = 232.079, *p* < 0.05, and η^2^ = 0.990; *H. verticillata*: F = 66.770, *p* < 0.05, and η^2^ = 0.965) resulting from varying lead concentrations can be observed in Figure 2b. For both *C. demersum* and *H. verticillata*, the SOD activity increased with an increasing concentration of lead, and the maximum activity occurred at 80 μM, which was 609% and 214%, respectively, of the levels in the controls. The results are consistent with the findings of Malar et al. [47] who observed a decline in the activity of SOD at a much higher lead concentration (approximately 4826 μM), which may be due to the inhibition of enzyme activity in the presence of excess H_2_O_2_. Compared to *H. verticillata*, the SOD activity in *C. demersum* was lower in the controls and higher in the 5~80 μM exposure groups, suggesting that the SOD activity in *C. demersum* had a stronger response to lead stress than in *H. verticillata*. Moreover, the SOD activity in *H. verticillata* in the 5–10 μM exposure groups exhibited no significant difference from the controls, indicating its insensitivity to the low lead concentrations.

Both CAT and POD can decompose H_2_O_2_—another toxic ROS produced mainly by the activity of SOD—directly into H_2_O and O_2_ in plant cells and play important roles in the main defense mechanism [48]. As shown in Figure 2c, the CAT activity of both plant species was significantly influenced by the lead concentration (*C. demersum*, F = 222.411, *p* < 0.05, and η^2^ = 0.989; *H. verticillata*, F = 162.170, *p* < 0.05, and η^2^ = 0.985). For *C. demersum*, the CAT activity increased at low concentrations of lead and then declined. The decline was also observed in other studies [47,49], which might result from inactivation of the CAT enzymes due to excess production of ROS. Regarding *H. verticillata*, the CAT activity kept increasing with an increasing lead concentration. A positive correlation (R^2^ = 0.94) between the CAT activity and increasing lead concentration was observed in the 0–20 μM exposure groups. The results suggest that CAT was continually activated as a defense mechanism to scavenge ROS, which agrees with the study by Asgari et al. [50]. However, the CAT activity did not exhibit significant changes at higher concentrations of lead (20~80 μM), indicating that *H. verticillata* may have reached the limit of its ability to synthesize CAT enzymes. In contrast to our results, a decline in CAT activity with an increasing heavy metal concentration was observed in *Lemna gibba* L. [51] and Brazilian elodea [13]. Furthermore, the CAT activity in *C. demersum* was higher than that in *H. verticillata* at low concentrations (0~10 μM Pb) and lower at high concentrations (20~80 μM Pb). The results suggest that CAT activity in *C. demersum* was more easily induced under low lead stress, while *H. verticillata* exhibited better ability to activate CAT and defend against lead toxicity when the lead concentration was greater than 10 μM.

The POD activity (Figure 2d) in the two plant species increased significantly with the increasing Pb concentration (*C. demersum*, F = 245.461, *p* < 0.05, η^2^ = 0.990; *H. verticillata*, F = 398.458, *p* < 0.05, η^2^ = 0.950) and reached a maximum at 80 μM. Positive correlations between the POD activity and lead concentration (*C. demersum*, R^2^ = 0.78; *H. verticillata*, R^2^ = 0.95) were observed for both plant species. The significantly enhanced POD activity in the plants exhibited a close association with plant adaptation to severe lead stress. POD activity induced by heavy metal stress has also been reported by Li et al. [52] in *Hibiscus cannabinus* L. The maximum activity of POD was 26.0 and 4.8 times, respectively, that of the control value. Clearly, the POD activity in *C. demersum* was much higher than that in *H. verticillata*, indicating a more positive response to lead toxicity.

### 3.4. PAL and PPO Enzyme Activities

**Figure 3:**
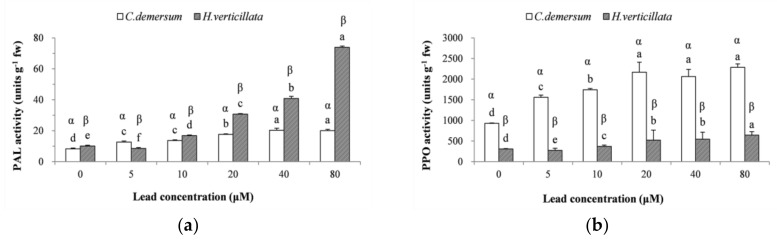
Effects of lead toxicity on (**a**) phenylalanine ammonia-lyase (PAL) and (**b**) polyphenol oxidase (PPO) in *C. demersum* and *H. verticillata.* All values are the mean of triplicate measurements ± SD (*n* = 3). ANOVA was considered significant at *p* < 0.05. Different Roman letters (a–f) and different Greek letters (α, β) indicate significant differences between concentrations for a plant species and significant differences between plant species for a concentration, respectively (DMRT, *p* < 0.05).

The significant effect (*C. demersum*, F = 114.482, *p* < 0.05, η^2^ = 0.979; *H. verticillata*, F = 324.888, *p* < 0.05, η^2^ = 0.993) of the lead concentration on PAL activity is presented in Figure 3a. The PAL activity in *C. demersum* increased gradually with the increasing lead concentration and reached a maximum at 40 μM. In *H. verticillata*, the PAL activity increased rapidly and reached a maximum at 80 μM Pb. The changes in PPO activity are presented in Figure 3b. The PPO activity in *C. demersum* and *H. verticillata* had a significant response to the lead concentration (*C. demersum*, F = 45.447, *p* < 0.05, η^2^ = 0.950; *H. verticillata*, F = 79.915, *p* < 0.05, η^2^ = 0.971). The POD activity of both plants increased gradually with the increasing Pb concentration.

We took PAL and PPO into consideration because they also perform defense-related functions. PAL, an all-important enzyme in phenylpropanoid metabolic pathways, can be used to reflect the plant defense capacity and synthesis rate of metabolites [53]. PPO plays a key role in the photosynthetic apparatus and can provide a foundation for heavy metal accumulation in plants [54]. Based on enzymatic assays, PAL and PPO activities in plants have been shown to be significantly induced by exposure to lead and are correlated with the plant species and concentration of lead. Similar results for other heavy metals have also been reported by Kováčik and Bačkor [55] and Ahsan et al. [56]. However, Song et al. [18] reported a different result: an initial increase followed by a decrease in the activity of PPO was observed in *H. verticillata* with increasing Ni concentration after 14 days of exposure, while the PAL maintained an increasing trend. Thus, the defense mechanisms in plants could lead to different responses to different heavy metals.

In this study, the PAL and PPO activities in both *C. demersum* and *H. verticillata* under lead stress exhibited significant differences compared to those in the controls, which indicates that a defense mechanism was activated in these plants. It should be noted that the PAL and PPO activities in both plant species exhibited the same increasing response with increasing lead concentration, whereas the activities of the two enzymes had the opposite trend. The PAL activity in *C. demersum* was weaker than in *H. verticillata*, while the PPO activity was stronger in *C. demersum* than in *H. verticillata*, which further indicates the different resistance mechanisms of the two plants. Moreover, for *C. demersum*, the PAL activities were not significantly different for the 40~80 μM groups, and the same was true for the PPO activities of the 20~80 μM groups, which suggests that the synthesis of the two enzymes were inhibited or hindered by high lead concentrations. However, significantly enhanced PAL and PPO activities were still observed in *H. verticillata* exposed to 80 μM Pb.

There exist both similarities and differences in the defense mechanisms of the two plant species. In *H. verticillata*, the link among the SOD, CAT, and POD activities is obvious based on their parallel changes. The three enzymes were significantly stimulated by the increasing lead concentration, indicating their roles in removing oxygen free radicals and maintaining the normal metabolic balance of cells under lead stress. The antioxidative enzymes also had the same performance in *C. demersum* except that a decrease was observed in the CAT activity at high concentrations of lead, indicating that the production mechanism of CAT might be disrupted under high lead stress. *C. demersum* contains higher levels of SOD, POD, and PPO. Constable and Ryan [57] suggested that sensitive plants usually exhibit an induction of protective metabolites under stress. A higher sensitivity to lead stress was observed in *C. demersum*, as indicated by the higher induction of the activities of SOD, POD, and PPO, as well as the inhibition of CAT and PAL activities at high lead concentrations. This effect might be an explanation for the significantly decreased biomass and protein content in *C. demersum*. Furthermore, the CAT activity was much lower than that of SOD and POD in both species, which is consistent with the findings of Li et al. [24], indicating that the activities of SOD and POD were more easily activated for defense in the antioxidant enzyme system in the submerged plants. The growth status and defense mechanism of *H. verticillata* were excellent after 14 days of exposure, suggesting a high tolerance potential, which needs to be verified by further study.

Apart from the enzymatic activities analyzed in this study, plants also possess other mechanisms to resist heavy metal stress. For instance, phytochelatins, a class of metal chelators, can bind metal ions to reduce toxic effects, and its binding efficiency shows an increasing trend with increasing heavy metal concentration [42,58]. Nonprotein thiols have also been proven to affect the ROS/NO balance in plants, thereby affecting the metal accumulation ability [59]. Therefore, it will be very interesting to analyze the responses of various defense mechanisms in plants in further studies. Furthermore, there always exist other types of pollutants in the wastewater, such as ammonia, which may affect the lead accumulation in plants, and this should also be studied.

## 4. Conclusions

In conclusion, both *C. demersum* and *H. verticillate* exhibited high potential to accumulate lead during their optimal performance period, and the lead accumulation ability of *C. demersum* was stronger. However, with an increase in the lead concentration, the increasing rate of lead accumulation in *C. demersum* gradually slowed and the growth status tended to be weaker. On the other hand, the lead accumulation in *H. verticillate* increased stably and the growth status was almost unaffected. That less damage was suffered by *H. verticillate* under the same lead concentration could also be demonstrated by the higher biomass and lower MDA content. Therefore, *H. verticillate* seemed to possess a stronger tolerance under the same lead stress, while *C. demersum* exhibited a much higher sensitivity. The excellent performance of the defense mechanism in *H. verticillate* might be related to the highly positive activity of CAT and PAL in the enzyme system. The other defense mechanisms in plants, such as phytochelatins, need to be further studied. The heavy metal accumulation ability and defense mechanisms of two pioneer, submerged plants during their optimal period provide criteria for the screening of hyperaccumulators.

## Figures and Tables

**Table 1:** Lead accumulation in *Ceratophyllum demersum* and *Hydrilla verticillata* exposed to different lead (Pb) concentrations for 14 days.

Concentration (μM)	Pb Accumulation (mg kg^−1^ dw)	
	*C. demersum*	*H. verticillata*
0	ND	ND
5	626.0 ^d^ ± 36.7	222.1 ^e^ ± 12.2
10	1510.0 ^c^ ± 135.0	305.6 ^d^ ± 19.4
20	1653.8 ^c^ ± 77.7	680.6 ^c^ ± 36.7
40	1992.9 ^b^ ± 110.3	1000.4 ^b^ ± 23.5
80	2462.7 ^a^ ± 58.4	1792.0 ^a^ ± 80.5

All values are the mean of triplicate measurements ± SD (*n* = 3). ANOVA was significant at *p* < 0.05. Different letters indicate significantly different values for a particular plant species (Duncan’s multiple range test (DMRT), *p* < 0.05).
